# Microwave magnetoimpedance and ferromagnetic resonance in Pr_0.6_Sr_0.4_MnO_3_

**DOI:** 10.1039/c9ra06786h

**Published:** 2019-09-17

**Authors:** A. Chanda, R. Mahendiran

**Affiliations:** Physics Department, National University of Singapore 2 Science Drive 3 Singapore-117551 Republic of Singapore phyrm@nus.edu.sg

## Abstract

We report the magnetic field dependence of electrical impedance (magnetoimpedance) of a ferromagnetic Pr_0.6_Sr_0.4_MnO_3_ sample carrying alternating current (ac) of frequency *f* = 1 MHz to 3 GHz measured using an impedance analyzer and broad band ferromagnetic resonance (*f* = 2 to 18 GHz) measured using a coplanar wave guide based spectrometer. Ac magnetoresistance is much larger than dc magnetoresistance and its sign at low magnetic fields changes from negative to positive with increasing frequency of the ac current. The field dependence of ac magnetoresistance shows a peak around *H*_dc_ = 0 for low frequencies but a double peak feature emerges at *H*_dc_ = ±*H*_p_ at higher frequencies and it shifts to higher magnetic field as the frequency of ac current increases. The field derivative of microwave power absorption measured by the broad band spectrometer shows features of ferromagnetic resonance and the resonance field increases with increasing frequency of microwave radiation following Kittel's equation for ferromagnetic resonance. A close correlation is found between the ferromagnetic resonance line shape and the positive peak in the ac magnetoresistance, which suggests the possibility of electrical detection of ferromagnetic resonance using high frequency current injected into a conducting magnetic sample.

## Abstract 2

We report magnetoimpedance of a ferromagnetic Pr_0.6_Sr_0.4_MnO_3_ sample carrying alternating current (ac) of frequency *f* = 1 MHz to 3 GHz measured using an impedance analyzer and also broad band ferromagnetic resonance (*f* = 2 to 18 GHz).

## Graphical abstract

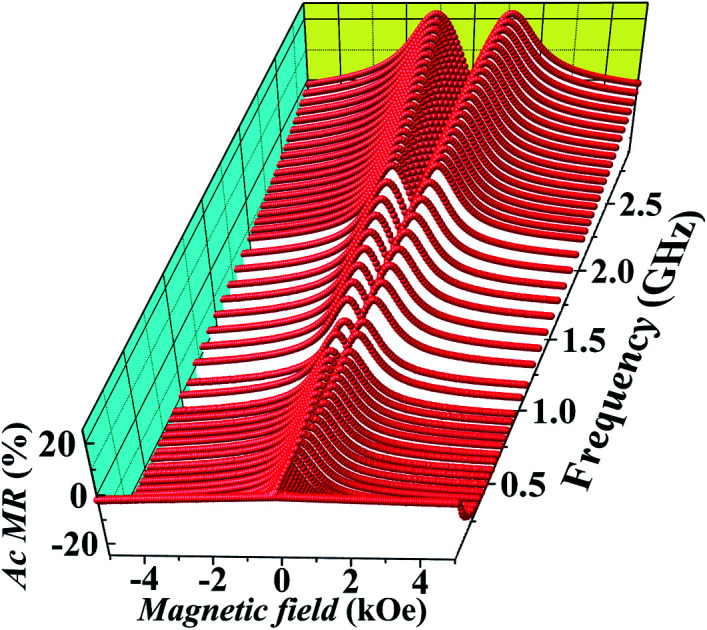

## 1. Introduction

The Mn-based perovskites known as manganites (R_1−*x*_A_*x*_MnO_3_ where R = La^3+^, Pr^3+^, *etc.*, and A = Sr^2+^, Ca^2+^*etc.*) have been extensively studied over the last three decades because of the two most exciting physical phenomena exhibited by them: colossal negative magnetoresistance for lighter rare earth ions (R = La, Pr, Nd)^1,2^ and spin-driven ferroelectricity in parent RMnO_3_ for heavy rare earth ions (R = Tb, Tm, Y).^3^ Parent compounds (RMnO_3_) possessing only Mn^3+^ ions are antiferromagnetic insulators whereas divalent cation substituted compounds containing mixed valent ions (Mn^3+^: t^3^_2g_e^1^_g_ and Mn^4+^: t^3^_2g_e^0^_g_) are ferromagnetic metals for *x* = 0.2–0.45 and antiferromagnetic insulators for 0.5 ≤ *x* ≤ 1 for R = La. The compositional range for ferromagnetism shrinks and ferromagnetic Curie temperature decreases as the ionic radius of R ion decreases. The influence of magnetic field on resistivity is greatest at the paramagnetic–ferromagnetic phase boundary. Majority of available studies focused on the magnetoresistance measured with a direct-current (dc) flowing in samples. Magnetoresistance in response to an alternating current (ac) through the sample barely received attention despite some claims of remarkable enhancement in the magnitude of ac magnetoresistance in the radio frequency regime (*f* = 1–15 MHz) compared to dc magnetoresistance in a field of few hundreds of Oersted.^4–8^ Such a large enhancement in the ac magnetoresistance with alternating currents has potential for practical applications compared to the dc magnetoresistance if the low-field sensitivity can be further improved.

Since hopping of the e_g_ electron between Mn^3+^(t^3^_2g_e^1^_g_) and Mn^4+^(t^3^_2g_e^0^_g_) *via* the intervening oxygen anion is responsible for electrical conduction in manganites, it is also of scientific curiosity to know how the resistivity and magnetoresistance are affected if the e_g_ electron is forced to oscillate at MHz and GHz frequencies. M. Dominguez *et al.*^9^ reported 70% magnetoresistance for 600 Oe near its Curie temperature (*T*_C_) in Nd_0.7_Sr_0.3_MnO_3_ thin film and V. Srinuvasu *et al.*^10^ found 80% magnetoresistance for a field of 600 Oe near *T*_C_ in La_0.7_Ba_0.3_MnO_3_ powder when these samples placed inside a microwave resonant cavity were irradiated with electromagnetic fields in microwave frequency range (*f* = 10 GHz). Microwave magnetoresistance was estimated from changes in microwave reflectivity of the cavity in absence and in presence of a dc magnetic field. In contrast to these reports, here, we report microwave magnetotransport in Pr_0.6_Sr_0.4_MnO_3_ by passing alternating current (ac) of frequency *f* through the sample and measuring its electrical impedance in presence of an external dc magnetic field.

Pr_0.6_Sr_0.4_MnO_3_ is ferromagnetic at room temperature (*T*_C_ = 305 K) but its magnetization shows a step-like change around *T*_S_ = 89 K (≪*T*_C_), which is apparently triggered by orthorhombic (*Pnma* space group) to monoclinic (*I*/2*a* space group) structural transition.^11,12^ Interestingly, this compound exhibits normal and inverse magnetocaloric effects at *T*_C_ and *T*_S_, respectively.^13^ A close correlation was also found between the magnetic entropy change, magnetothermopower and magnetization. While dc resistivity showed a very weak anomaly at *T*_S_ in a single crystalline sample,^14^ it was not detectable in the polycrystalline sample. However, ac resistance (1 MHz < *f* < 5 MHz) showed a pronounced anomaly at *T*_S_. It is of our interest to understand the behavior of magnetoresistance at frequencies higher than 5 MHz. Here, we extend the magnetotransport measurement in Pr_0.6_Sr_0.4_MnO_3_ over a wide frequency range (*f* = 1 MHz to 3 GHz) in response to radio frequency current flowing through the sample. In addition, we also report microwave absorption for multiple frequencies (*f* = 2 to 10 GHz) of microwave electromagnetic field. Our results indicate that high frequency magnetoimpedance shows features of ferromagnetic resonance (FMR). Although FMR and electron spin resonance (ESR) in K- and Na-doped Pr_0.6_Sr_0.4_MnO_3_ (ref. 15) as well as other manganites^16–18^ have been studied using conventional MW cavity resonators, FMR excited by MW current flowing through the sample has not been investigated. Here, we demonstrate a broadband detection of FMR in Pr_0.6_Sr_0.4_MnO_3_ by electrical means using an impedance analyzer. The main advantage of our magnetoimpedance (MI) method is the broad operational frequency range unlike the conventional MW cavity resonator based technique which usually operates at a fixed frequency (around 9.8 GHz) in X-band. Because the MI technique probes spin dynamics, it can be exploited to obtain Gilbert's damping parameter, which is not possible with a single frequency cavity resonator FMR technique.

## 2. Experimental details

**Fig. 1:**
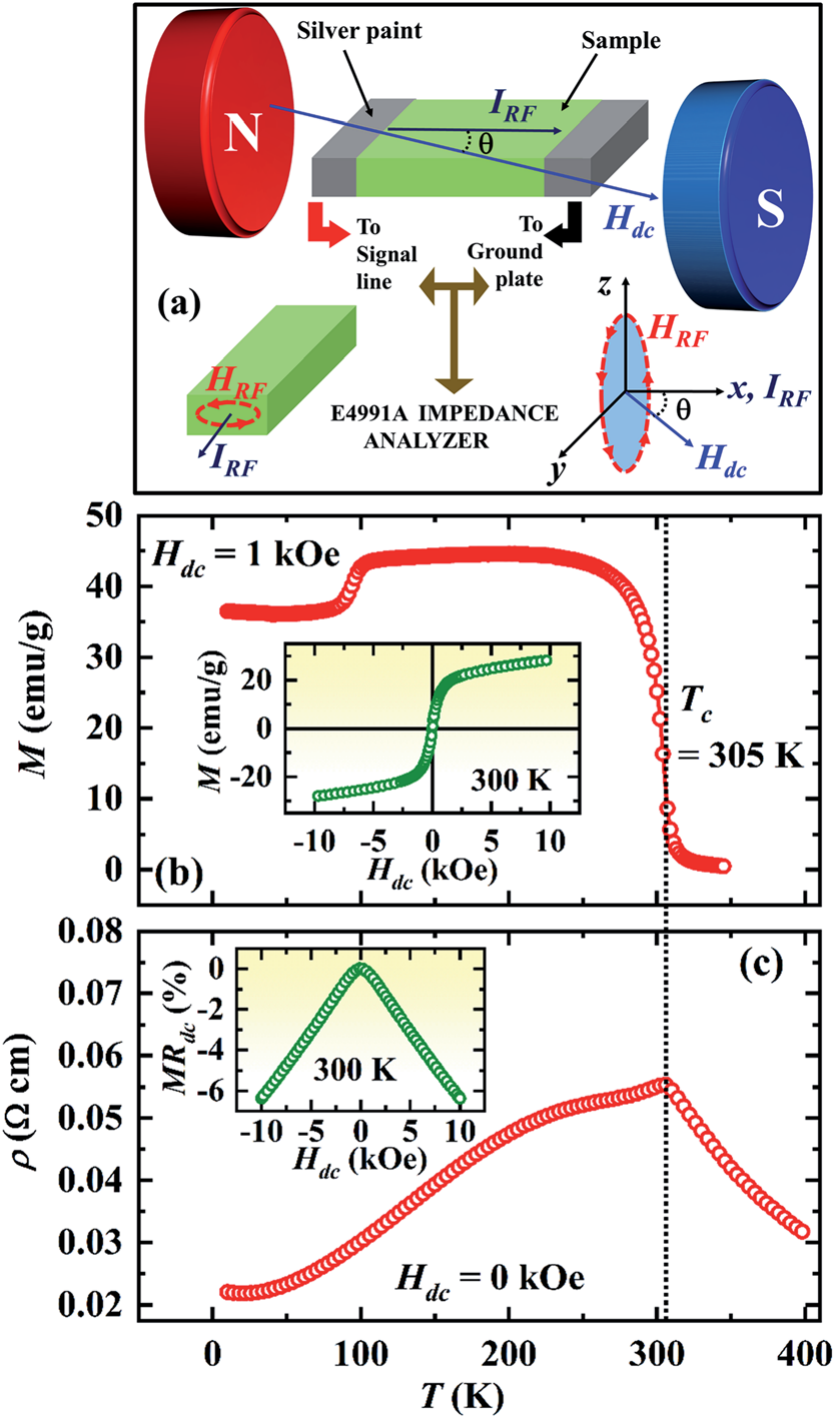
(a) A schematic illustration of the broadband magnetoimpedance experimental configuration. (b) Temperature dependence of magnetization, *M*(*T*) of Pr_0.6_Sr_0.4_MnO_3_ in a magnetic field of *H*_dc_ = 1 kOe, inset shows *M*(*H*) isotherm at 300 K (c) temperature dependence of four-probe dc resistivity of Pr_0.6_Sr_0.4_MnO_3_, inset shows the dc magnetoresistance, MR_dc_ at 300 K.

Polycrystalline sample of Pr_0.6_Sr_0.4_MnO_3_ was prepared by conventional solid state reaction method. It crystallizes in single phase with orthorhombic structure (space group *Pnma*) at room temperature.^19^ Magnetization was measured using a vibrating sample magnetometer (VSM) and four probe electrical resistivity was measured in a Physical Property Measuring System (PPMS). The sample was cut into a rectangular plate of dimensions 5 mm × 3 mm × 2 mm for both dc resistivity and magnetoimpedance measurement. A radio frequency impedance analyzer (Agilent E4991A) was used to measure the resistive (*R*) and reactive (*X*) components of the complex impedance, *Z*(*f*) = *R*(*f*) + i*X*(*f*) of the sample by employing the radio frequency (rf) current–voltage method. As shown in the Fig. 1(a), the sample placed on an Agilent test probe stage is attached to the signal line at one end and the other end of the sample is connected to the ground plate using silver paint. To avoid electrical contact between the sample surface and the ground plate, a layer of kapton tape was inserted between them. The rf current flows through the sample from the signal line to the ground plane. The test probe stage is placed at the center of an electromagnet. We define the angle between the direction of rf current flow and that of the dc magnetic field (*H*_dc_) produced by the electromagnet by *θ*. The *R* and *X* at selected frequencies were measured while sweeping *H*_dc_ for *θ* = 0°, 30°, 60° and 90°. We define dc magnetoresistance as MR_dc_ = [*ρ*(*H*) − *ρ*(*H* = 0)]/*ρ*(*H* = 0) × 100%, where, *ρ* is the dc electrical resistivity of the sample. AC magnetoresistance is defined as MR_ac_ = [*R*(*H*, *f*) − *R*(*H* = 0, *f*)]/*R*(*H* = 0, *f*) × 100% for a frequency *f* of alternating current and the magnetoreactance is defined as MX = [*X*(*H*, *f*) − *X*(*H* = 0, *f*)]/*X*(*H* = 0, *f*) × 100%. We also define magnetoimpedance as, MZ = [*Z*(*H*, *f*) − *Z*(*H* = 0, *f*)]/*Z*(*H* = 0, *f*) × 100%, where, *Z* = (*R*^2^ + *X*^2^)^1/2^. The magnetic field dependence of the field derivative of microwave power absorption (d*P*/d*H*) was measured using a commercial broadband ferromagnetic resonance spectrometer (NanOsc Phase-FMR from Quantum Design Inc.) that makes use of coplanar waveguide method. The PPMS was used to provide an in-plane dc magnetic field (*H*_dc_) in such a way that the rf magnetic field (*H*_rf_) generated in the waveguide is perpendicular to *H*_dc_ (see Fig. 4(a)). The dc magnetic field was modulated using a pair of Helmholtz coils, which provided low amplitude (1 Oe) and low frequency (440 Hz) ac magnetic field (*H*_ac_). Because of the field modulation and the lock-in technique used, microwave power absorption was recorded as the field derivative (d*P*/d*H*) as like in conventional ESR spectrometers.

## 3. Results

The main panel of Fig. 1(b) shows *M*(*T*) of Pr_0.6_Sr_0.4_MnO_3_ sample in a dc magnetic field of *H*_dc_ = 1 kOe while cooling from *T* = 350 K. The ferromagnetic Curie temperature (*T*_C_) determined from the minimum of d*M*/d*T* curve is 305 K. The inset of Fig. 1(b) shows the magnetic field dependence of magnetization at *T* = 300 K which confirms the soft ferromagnetic nature of the sample. The temperature dependence of dc resistivity *ρ*(*T*) (see the main panel of Fig. 1(c)) measured in zero external magnetic field shows a change from thermally activated behavior in the paramagnetic state to metallic behavior below *T*_C_ with a peak occurring at *T*_C_. The dc magnetoresistance (MR_dc_) at *T* = 300 K (inset of Fig. 1(c)) shows a single peak at *H*_dc_ = 0 with a negative sign and increases in magnitude. The absolute value of MR_dc_ is 6% at *H*_dc_ = ±10 kOe.

**Fig. 2:**
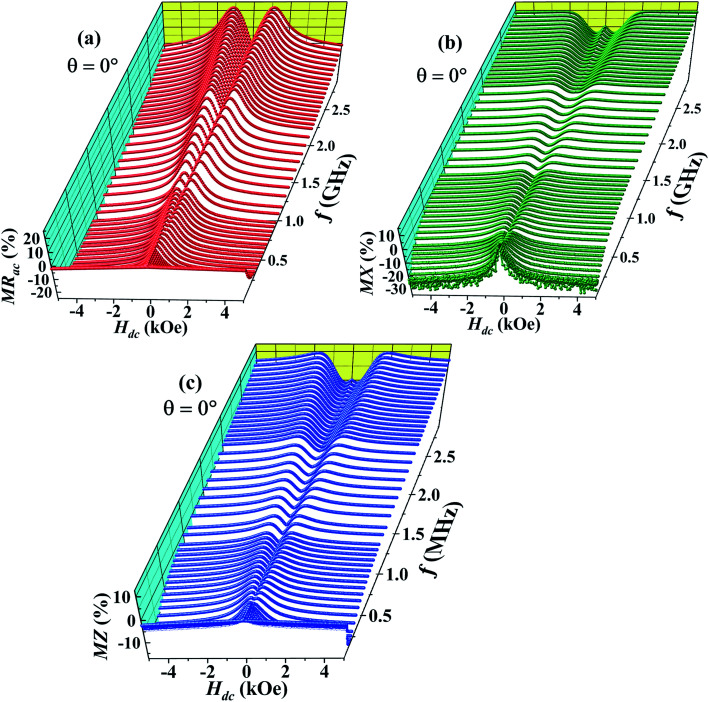
Magnetic field dependence of (a) ac magnetoresistance (MR_ac_) (b) magnetoreactance (MX) and (c) magnetoimpedance (MZ) as a percentage change at *T* = 300 K for *θ* = 0° for different frequencies of the ac current between *f* = 1 MHz and 3 GHz.

Fig. 2(a) shows the magnetic field dependence of the ac magnetoresistance (MR_ac_) at room temperature for different frequencies of the alternating current (*f* = 1 MHz to 3 GHz). The dc magnetic field (*H*_dc_) produced by the electromagnet is parallel to the direction of rf current (*I*_rf_) passing through the sample (*θ* = 0°). MR_ac_ is negative at *f* = 1 MHz with an absolute value of 2% at *H*_dc_ = 5 kOe and shows a single peak at *H*_dc_ = 0. As the frequency of the alternating current increases, the absolute value of MR_ac_ increases up to a certain frequency (∼500 MHz) but decreases for still higher frequencies. Above *f* = 500 MHz, the single peak at *H*_dc_ = 0 splits into two symmetrical peaks at *H*_dc_ = ±*H*_p_ on either sides of the zero field and both these peaks migrate towards higher magnetic fields as *f* increases further. The sample shows a positive ac MR in the field range −*H*_p_ ≤ *H*_dc_ ≤ +*H*_p_ for *f* > 500 MHz.

Fig. 2(b) shows the magnetic field dependence of the magnetoreactance (MX). MX also shows double peaks at *H*_dc_ = ±*H*_q_(*H*_q_ > *H*_p_) above 500 MHz which also move towards higher field with increasing frequency. The double peaks in MX at ±*H*_q_, unlike in MR_ac_, fade above *f* = 1 GHz. In addition to the double peaks, MX also shows a single peak centered at origin (*H*_dc_ = 0) above 2 GHz culminating in double dips between *H*_dc_ = 0 and ±*H*_q_. The field dependence of magnetoimpedance (MZ) shown in Fig. 2(c) is almost identical to that of MX.

**Fig. 3:**
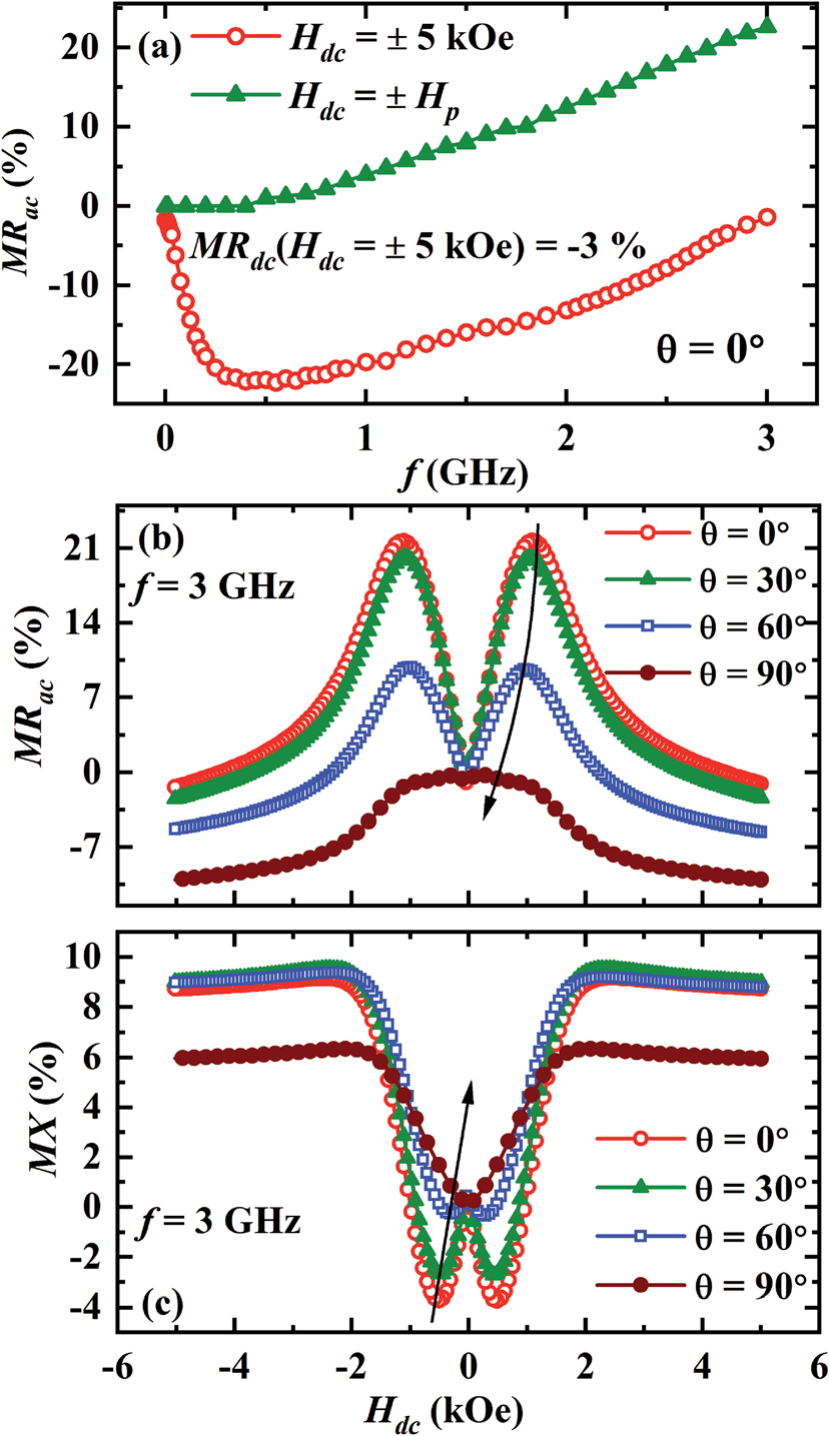
(a) The ac MR as a function of frequency at room temperature for *θ* = 0°, magnetic field dependence of (b) MR_ac_ and (c) MX for *θ* = 0°, 30°, 60° and 90° at a fixed frequency *f* = 3 GHz.

The main panel of Fig. 3(a) displays the frequency dependence of MR_ac_ at the highest field for *θ* = 0°. The absolute value of negative ac MR increases with increasing frequency and becomes maximum around 500 MHz (∼22%) but decreases towards zero with further increasing frequency. On the other hand, the value of positive ac MR at *H*_dc_ = ±*H*_p_ increases gradually with increasing frequency and reaches ∼23% (see the inset of Fig. 3(a)) for *f* = 3 GHz. It is noteworthy that the value of MR_dc_ at 5 kOe is only 3% which is almost 7 times lower than the value of MR_ac_ at the same field for *f* = 500 MHz.

We have also studied how the observed anomalous features vary if the angle between radio frequency (rf) current direction and the direction of dc magnetic field (*θ*) is changed. MR_ac_ and MX for *θ* = 0°, 30°, 60° and 90° are depicted in the Fig. 3(b) and (c), respectively for *f* = 3 GHz. It is clear that the intensity of the double peaks at *H*_dc_ = ±*H*_p_ in MR_ac_ as well as the double dips at *H*_dc_ = ±*H*_q_ in MX decreases with increasing *θ*. Moreover, the double peak (dip) positions in MR_ac_ (MX) also shift to lower fields as *θ* increases and they disappear completely for *θ* = 90°.

**Fig. 4:**
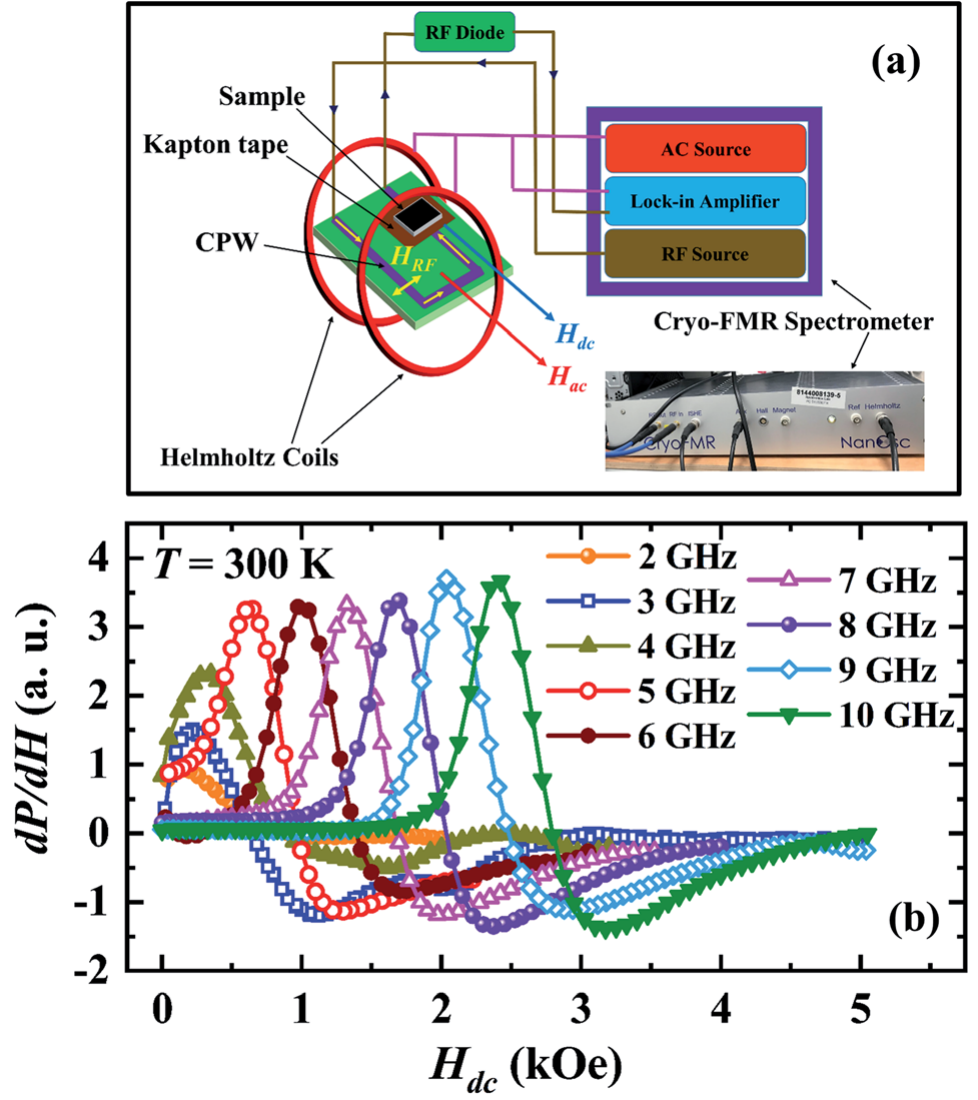
(a) A schematic representation of the broadband FMR setup, (b) magnetic field dependence of the field derivative of the microwave power absorption (d*P*/d*H*) at *T* = 300 K at selected fixed frequencies of the microwave magnetic field (*f* = 2 to 10 GHz).

Fig. 4(b) shows the magnetic field dependence of microwave power absorption recorded as the field derivative (d*P*/d*H*) for different frequencies of the microwave magnetic field between *f* = 2–10 GHz at room temperature. The microwave absorption signal for each frequency exhibits a magnetic resonance feature a dip at a higher field followed by a peak at a lower field when the dc field is reduced from a high value to zero. The signal for 2 GHz shows a peak around *H*_dc_ = 160 Oe. The peak in d*P*/d*H* shifts towards higher *H*_dc_ as the frequency of the microwave electromagnetic field increases similar to the behavior of ac MR.

**Fig. 5:**
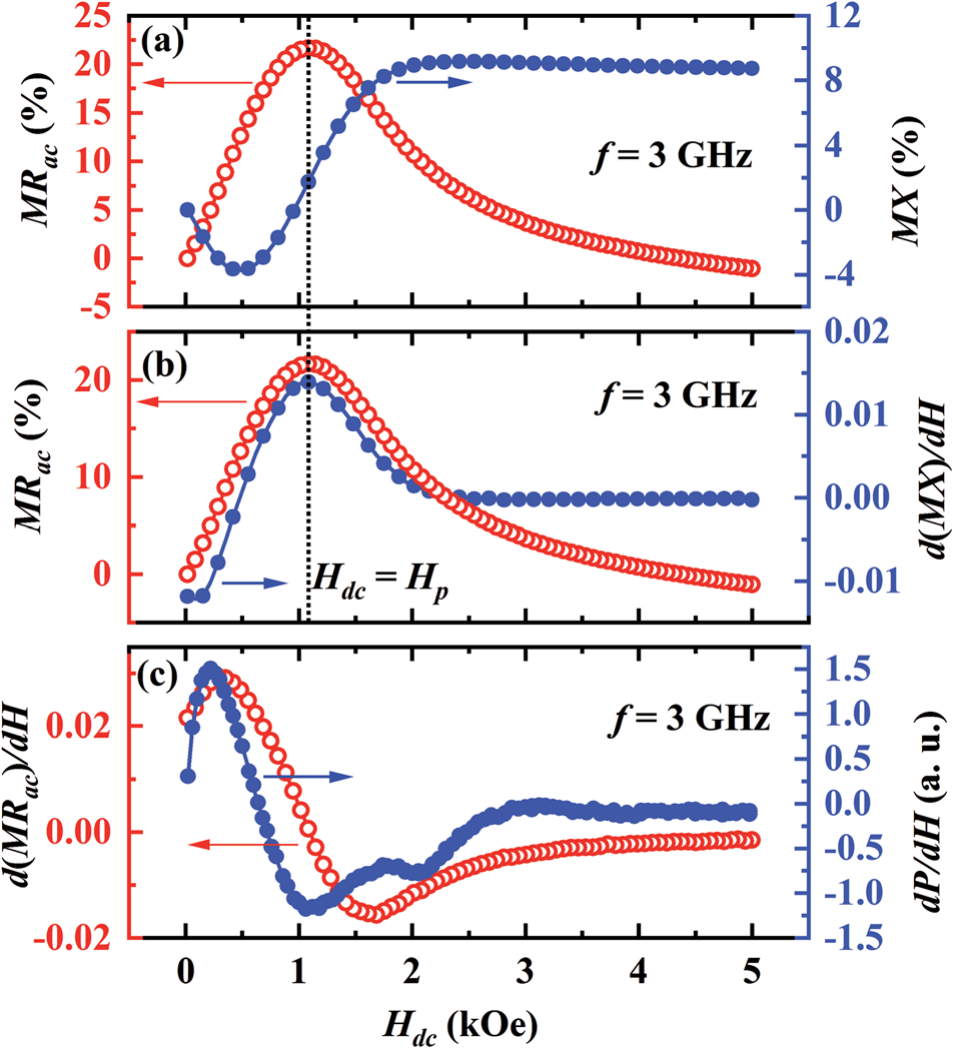
(a) MR_ac_ on the left scale and MX on the right scale, (b) MR_ac_ on the left *y* scale and first derivative of the MX(d(MX)/d*H*) on the right scale, (c) first derivative of the MR_ac_(d(MR_ac_)/d*H*) on the left *y* scale and (d*P*/d*H*) on the right scale for *f* = 3 GHz at *T* = 300 K.

To understand a possible link between the anomalous behaviors of MR_ac_ and MX, we plot the MR_ac_ (left scale) and the MX (right scale) together in a single graph for *f* = 3 GHz in Fig. 5(a). A careful look reveals that the peak in MR_ac_ isotherm almost coincides with the point of inflection in MX isotherm but MX is nearly independent of *H*_dc_ above 2 kOe unlike that of the MR_ac_. It is clear from the Fig. 5(b) that the peak in MR_ac_ (left *y*-axis) coincides with the peak in the field derivative of MX (right *y*-axis). In Fig. 5(c), we plot the field derivative of the MR_ac_ on the left *y*-axis and d*P*/d*H* on the right *y*-axis for *f* = 3 GHz. It is evident that the peak positions of d(MR_ac_)/d*H* and d*P*/d*H* closely match with each other. However, the linewidth of d(MR_ac_)/d*H* is higher than that of d*P*/d*H*.

## 4. Discussion

We summarize important observations from the above results as follows: (1) Pr_0.6_Sr_0.4_MnO_3_ is a room temperature ferromagnet with *T*_C_ = 305 K. (2) The ac magnetoresistance MR_ac_ is negative and shows a peak at *H*_dc_ = 0 for *f* ≤ 500 MHz but exhibits positive double peaks at *H*_dc_ = ±*H*_*p*_ for higher frequencies. (3) The absolute value of negative ac MR at *H*_dc_ = 5 kOe increases with frequency and becomes maximum (∼22%) at *f* = 500 MHz which is significantly higher than the value of dc MR (∼−3%) at the same field. (4) The value of positive MR_ac_ at *H*_dc_ = ±*H*_p_ increases with frequency and becomes ∼+23% for *f* = 3 GHz. (5) As *θ* increases from resonant (0°) to non-resonant (90°) configuration, the double peak at *H*_dc_ = ±*H*_p_ in the MR_ac_ shifts towards the origin as well as the value of MR_ac_ at *H*_dc_ = ±*H*_p_ decreases considerably. (6) The magnetic field corresponding to the peak in MR_ac_ isotherm coincides with the point of inflection of MX isotherm. (7) The d*P*/d*H* shows a Lorentzian line shape and the maximum in d*P*/d*H* shifts towards higher *H*_dc_ with increasing frequency. (8) The peak positions in d(MR_ac_)/d*H* and d*P*/d*H* closely match with each other.

Let us first shed some light on the origin of such anomalous behavior of the ac magnetotransport in this sample. The application of a dc electric field to the sample forces the mobile e_g_ electron to hop between Mn^3+^ and Mn^4+^ ions in the background of immobile t^3^_2g_ core electrons. The hopping is dependent on the relative angle between t_2g_ spins of Mn ions. While dc or low frequency ac current flows uniformly throughout the volume of the sample, high frequency current tends to flow only in thin surface layer of thickness “*δ*” due to skin effect. The “skin depth” *δ* decreases with increasing angular frequency (*ω* = 2π*f*) of the ac current and dependent on dc resistivity (*ρ*) and transverse magnetic permeability (*μ*_t_) of the sample through the expression, 
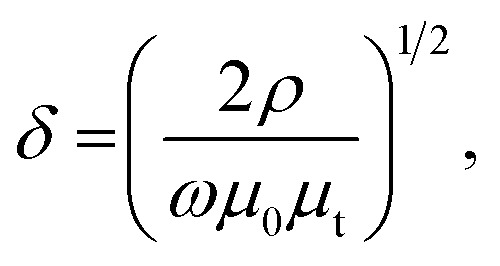
 where, *μ*_0_ is the free space permeability. Instead of resistance for dc current, we consider impedance for alternating current. The electrical impedance of a rectangular slab with thickness 2*t* and infinite width is given by,^20^

1 *Z* = *R*_dc_*kt* coth (*kt*)

where, *k* = (1 + *i*)/*δ* is the wave vector and *R*_dc_ is the dc resistance of the sample. Using *ρ* = 55 × 10^−5^ Ω m at 300 K and taking *μ*_t_ = 1 corresponding to the non-magnetic limit, the value of *δ* in our sample at *f* = 3 GHz is 220 μm which is almost 10 times smaller than the sample thickness (2 mm). When the skin effect is very strong, *i.e.*, *δ* ≪ 2*t*, *kt* ≫ 1 and hence, coth (*kt*) → 1. In that case, the expression for surface impedance of the sample *Z* becomes,^20^2
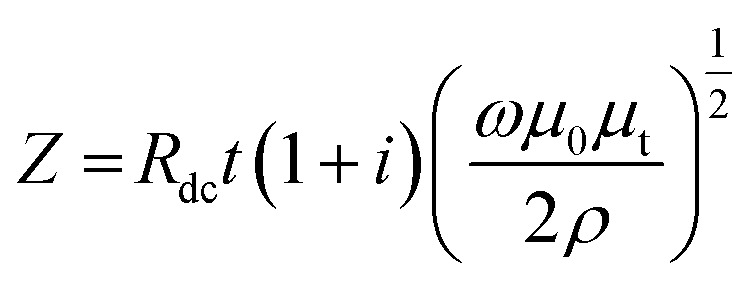


Besides the skin effect, the flow of radio frequency (rf) current in the sample generates a transverse circular rf magnetic field (*H*_rf_) which interacts with the magnetization of the sample and hence, affects the transverse component of the magnetic permeability (*μ*_t_). The transverse permeability is a complex quantity and given by 
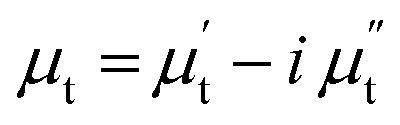
 where, 
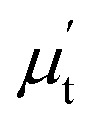
 is the in-phase component (magnetization is in-phase with the rf magnetic field) and 
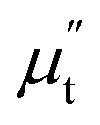
 is the out-of-phase component (magnetization is not in-phase with the rf magnetic field).

Hence, the complex surface impedance is given by,^21^3
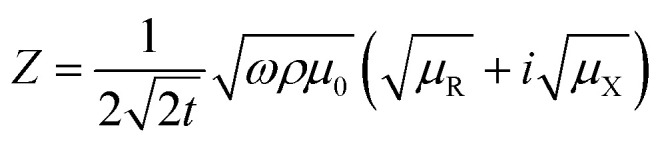
where, 
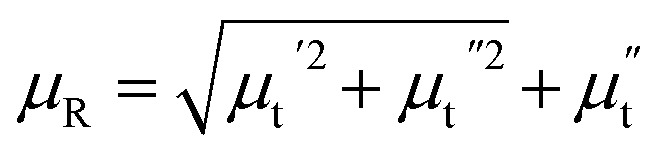
 and 
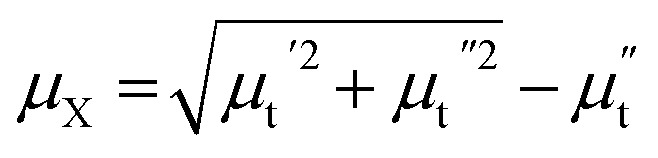
.

Hence, the real (R) and imaginary (X) parts of the complex impedance are 
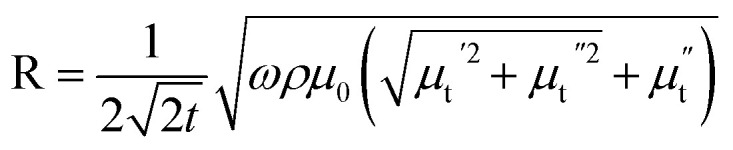
 and
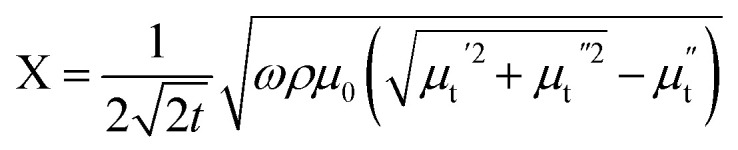
, respectively. Since *μ*_t_ is affected by external dc magnetic field, it influences the field dependence of both R and X. So, unlike dc magnetoresistance, ac magnetoresistance in the high frequency regime is dominated mostly by magnetization dynamics of the sample rather than changes in scattering rate of the conducting electrons.

When the applied dc magnetic field (*H*_dc_) is smaller than the saturation field, *μ*_t_ of the sample depends on the relative alignment of the magnetic easy axis and the direction of *H*_dc_. If *H*_dc_ is parallel to the easy axis, *μ*_t_ decreases monotonically from its maximum value in zero field to nonmagnetic limit (*μ*_t_ = 1) with increasing strength of the dc magnetic field. However, if *H*_dc_ is perpendicular to the magnetic easy axis, magnetization of the sample rotates but does not switch towards *H*_dc_ unless *H*_dc_ exceeds the anisotropy field *H*_k_. The transverse permeability is expected to diverge at *H* = *H*_k_ for an ideal system but usually shows a peak at *H*_k_ in non-ideal case,^20–22^ Since, 
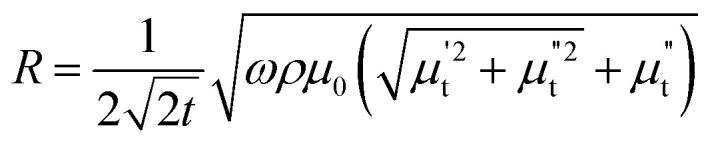
, the ac resistance or surface resistance increases with *H*_dc_ and shows a peak at *H*_dc_ = ±*H*_p_, where *H*_p_ is close to *H*_k_, which explains the appearance of double peaks in MR_ac_ of our sample in the frequency range *f* = 500–1000 MHz for the orthogonal configuration of *H*_dc_ and *H*_rf_ (*θ* = 0°). The disappearance of double peak behaviour in MR_ac_ for *θ* = 90° is expected because *μ*_t_ will decrease monotonically with *H*_dc_ for the parallel configuration of *H*_dc_ and *H*_rf_. The shift of R-peak at *H*_dc_ = ±*H*_p_ with frequency is small in the frequency regime *f* = 500–1000 MHz which could be due to dispersion in the anisotropy field (only few tens of Oe).^23^ However, gyromagnetic effect, *i.e.*, precession of magnetization about the direction of dc magnetic field dominates at frequencies higher than 500 MHz. When the frequency of magnetization precession matches with that of the rf magnetic field, steady state precession of magnetization, *i.e.*, ferromagnetic resonance (FMR) occurs in the sample. The FMR frequency *f*_res_ for a thin plate like sample increases with the frequency following the Kittel's expression when *H*_dc_ is perpendicular to *H*_rf_,^24^4
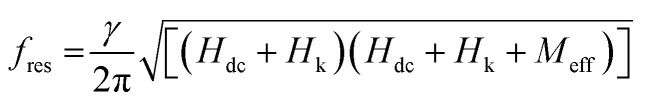
where, *H*_k_ is the transverse anisotropy field, *M*_eff_ is the effective magnetization whose value is close to saturation magnetization (*M*_S_) and *γ* is the gyromagnetic ratio, *γ* = *gμ*_B_/*ħ*, where “*g*” is the “Lande' *g*-factor”.

Britel *et al.*, showed that a close link exists between the magnetoimpedance measured by electrical method and the FMR recorded using a microwave electron spin resonance (ESR) spectrometer.^25^ In an ESR spectrometer, a sample placed inside a microwave (MW) resonant cavity is irradiated with electromagnetic field in microwave range, usually 9–10 GHz. The magnetic component of electromagnetic field impinging on the sample inside MW cavity is transverse to the dc magnetic field produced externally by an electromagnet. Similarly, the rf/MW magnetic field generated by rf/MW current in the sample is also transverse to the dc magnetic field in our magnetoimpedance measurement when *θ* = 0°. The sample absorbs maximum power from the MW electromagnetic field during FMR. The power absorption (*P*) in the sample per unit volume is related to the out-of-phase component of transverse permeability through the expression5
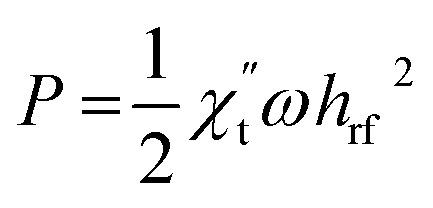
where, *h*_rf_ is the amplitude of the alternating magnetic field (*H*_rf_(*t*) = *H*_0_ + *h*_rf_ cos *ωt*), 
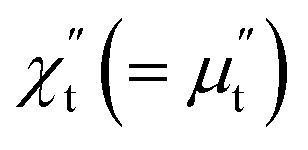
 is the out-of-phase component of transverse susceptibility. 
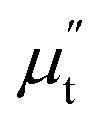
 goes through a maximum at the resonance field (*H*_res_) and hence, *P*(*H*_dc_) also shows maximum at *H*_dc_ = *H*_res_. Since 
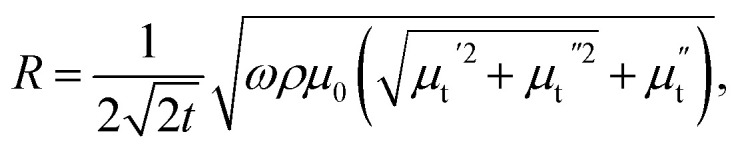
 the appearance of peak in MR_ac_ at *H*_dc_ = ±*H*_p_ can be considered as a consequence of the maximum power absorption by the sample due to FMR. Similar behavior was reported in magnetic field dependence of impedance (*Z*) for Fe–Co–Si–B amorphous ribbons^26^ where the field corresponding to the peak in real component of *Z* shifts rapidly to higher fields with increasing frequency. D. de Cos *et al.*,^27^ also found a close correlation between d*R*_ac_/d*H* obtained from magnetoimpedance and d*P*/d*H* obtained from microwave power absorption in cavity resonator.

The magnetic field dependence of power absorption, *P*(*H*_dc_) usually shows a Lorentzian line shape described by the expression,^28^6
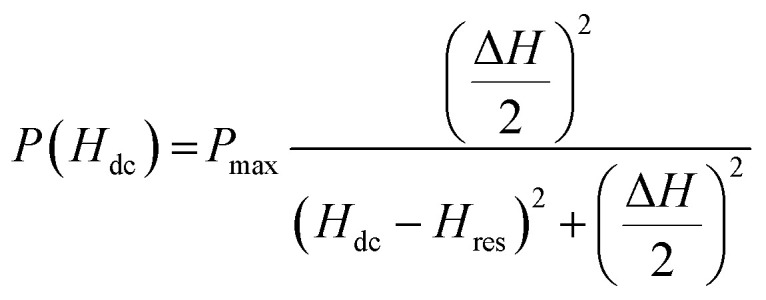
where, *P*_max_ is the absorbed power at the resonance field (*H*_dc_ = *H*_res_) and Δ*H* is the full line width at half the peak power (FWHM). Since the magnetic field dependence of *R* reflects the behavior of *P*(*H*_dc_), the field dependence of the high frequency MR_ac_ can also be described by the Lorentzian function. We have fitted the MR_ac_ line shapes with the following equation,^29^7
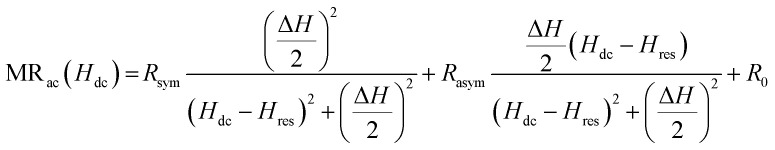
where, *R*_sym_ and *R*_asym_ are the coefficients of symmetric and antisymmetric Lorentzian functions, *H*_res_ and Δ*H* are the resonance field and linewidth, respectively for the MR_ac_ line shape and *R*_0_ is a constant offset parameter. Eqn (7) is composed of a symmetric and an antisymmetric Lorentzian function which is generally used for the electrical detection of FMR.^29,30^ The symmetry of FMR line shape depends on the relative phase between microwave electric and magnetic field components.^29^ The symmetric line shape accounts for the contribution of rf current which is in-phase with the rf magnetization and the antisymmetric line shape accounts for the out-of-phase contribution.^31^ Thus, the resultant line shape is a linear combination of symmetric and antisymmetric contributions which leads to the asymmetric nature of the FMR line shape.^29^

**Fig. 6:**
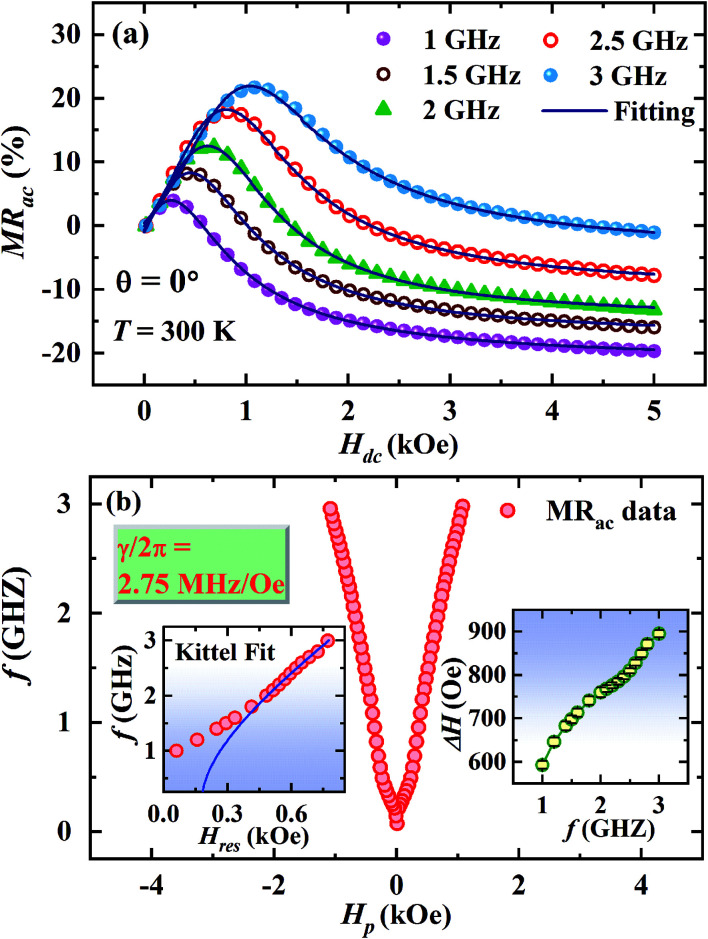
(a) Fitting of MR_ac_ line shapes for *θ* = 0°. (b) Main panel: a plot of frequency (*f*) as a function of the dc magnetic field (*H*_p_) at which the ac MR shows a peak, left inset shows Kittel-fit to the plot of *f vs.* resonance field (*H*_res_) extracted from the MR_ac_ data and right inset shows frequency dependence of linewidth (Δ*H*).

Fig. 6(a) displays the fitting of the MR_ac_ line shape with the eqn (7) for a few selected frequencies between *f* = 1 and 3 GHz. The main panel of Fig. 6(b) shows *H*_dc_ corresponding to the peak in MR_ac_ on *x*-scale and the corresponding frequency on *y*-scale which clearly indicates the evolution of double peaks and their rapid upshifting in *H*_dc_ with increasing frequency of ac current. The plot *f vs.* resonance field (*H*_res_) above 1 GHz obtained from the fitting of the MR_ac_ line shape are shown in the left inset of Fig. 6(b). Fitting of the *f vs. H*_res_ curve with the Kittel's equation for FMR described by eqn (4) yields *γ*/2π = 2.75 MHz Oe^−1^ and hence, *g* = 1.9657 which is slightly lower than the theoretically expected value of *g* = 2 for ferromagnetic manganites.^32^ Belmeguenai *et al.*^33^ obtained *g* = 1.95 in La_0.7_Sr_0.3_MnO_3_ thin films using the microstrip FMR (MS-FMR) technique. In order to obtain an accurate value of *g* factor, measurements need to be extended to several tens of GHz. As shown in the right inset of Fig. 6(b), Δ*H* increases non-linearly with frequency in the low frequency region but linearly in the high frequency regime. We have also extracted the values of 4π*M*_S_ and *H*_k_ from the fitting which are 1900 ± 70 Oe and 190 ± 20 Oe, respectively.

**Fig. 7:**
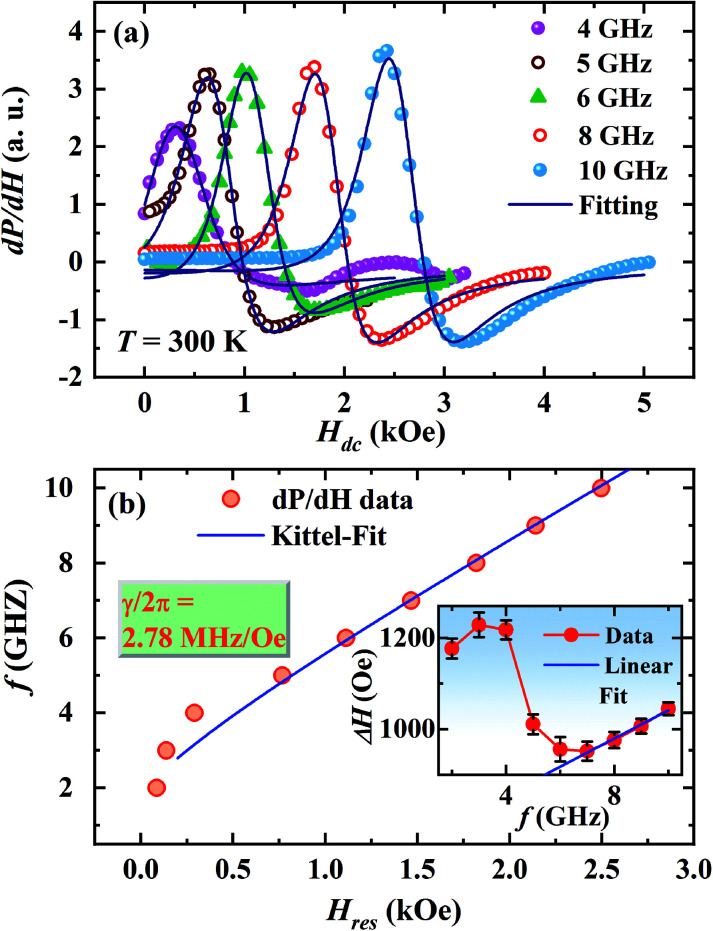
(a) Fitting of d*P*/d*H* line shapes at *T* = 300 K for different frequencies, (b) main panel shows the Kittel-fit to the plot of frequency (*f*) *vs.* resonance field (*H*_res_) extracted from d*P*/d*H* data and the inset shows frequency dependence of Δ*H* with a linear fit for *f* > 6 GHz.

In order to confirm whether the observed anomalous behaviour of the MR_ac_ corresponds to FMR, we have fitted the field derivative of the power absorption (d*P*/d*H*) by a combined function containing derivatives of symmetric and antisymmetric Lorentzian functions as^34^8
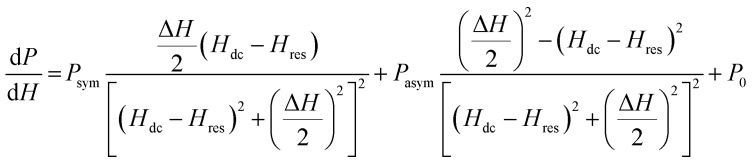
where, *P*_sym_ and *P*_asym_ are the coefficients of symmetric and antisymmetric Lorentzian derivatives, *H*_res_ and Δ*H* are the resonance field and linewidth (FWHM), respectively for the d*P*/d*H* line shape and *P*_0_ is the constant offset parameter. Fig. 7(a) displays the fitting of d*P*/d*H* line shape with the eqn (8) for selected frequencies between *f* = 2 and 10 GHz. The plot of frequency (*f*) *vs.* resonance field (*H*_res_) obtained from the fitting of d*P*/d*H* line shapes are shown in main panel of Fig. 7(b). Fitting of the *f vs. H*_res_ curve with eqn (4) yields *γ*/2π = 2.78 MHz Oe^−1^ and hence, *g* = 1.987 which is close to the value of *g*-factor extracted from the fitting of ac MR line shape.

The extracted the values of 4π*M*_S_ and *H*_k_ from the fitting of d*P*/d*H* line shape are 2200 ± 100 Oe and 190 ± 30 Oe, respectively. It is to be noted that the value of *H*_k_ obtained from both magnetoimpedance and microwave absorption measurements are close to *H*_k_ = 200 ± 10 Oe estimated from the fitting of *M*(*H*) isotherm with the law of approach to saturation model which is expressed as,^35^9
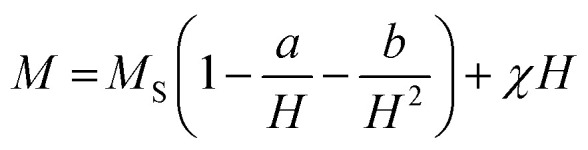
where, the coefficient *a* is related to micro-stress and the coefficient *b* is connected to the first order magnetocrystalline anisotropy coefficient *K* through the expression, 
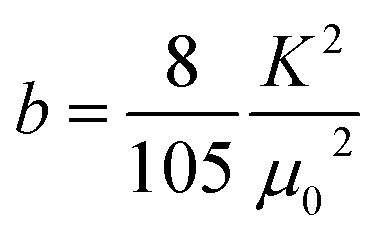
. The anisotropy field *H*_k_ can be estimated from the relation: *H*_k_ = 2*K*/*μ*_0_*M*_S_. The frequency dependence of Δ*H* extracted from the fitting of d*P*/d*H* curve is shown in the inset of Fig. 7(b) which clearly shows a nonlinear dependence below 6 GHz but a linear dependence for higher frequencies.

Nonlinear *f*-dependence of Δ*H* was found in various magnetic nanostructures which was attributed to the presence of two relaxation processes: the intrinsic Gilbert's damping and the two-magnon scattering.^36^ The two-magnon scattering is an extrinsic damping mechanism which causes inhomogeneous broadening of Δ*H* and responsible for damping enhancement. We have fitted the *f* dependence of Δ*H* above 6 GHz with the following equation,^37^10
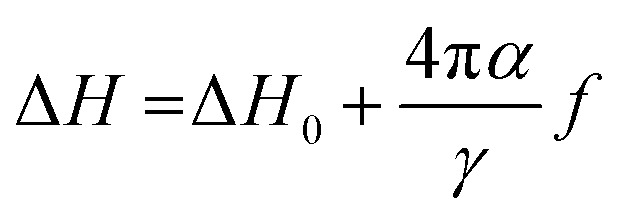
where, Δ*H*_0_ accounts for the extrinsic damping contributions and *α* is the Gilbert's damping parameter. The value of *α* extracted from the fitting is 13.6 × 10^−3^ ± 0.8 × 10^−3^ and it is higher than the value found in La_0.7_Sr_0.3_MnO_3_/NGO thin film (*α* = 7.8 × 10^−4^)^38^ and Pt capped La_0.7_Sr_0.3_MnO_3_/STO thin film (*α* = 5.93 × 10^−3^).^39^ A possible reason for the large value of *α* observed could be due to polycrystalline nature of our sample. We need to investigate spin dynamics in epitaxial thin films by this magnetoimpedance technique in future.

In recent years, electrical detection of FMR using microstrip (MS) or co-planar waveguide (CPW) is gaining popularity to investigate spin dynamics in magnetic nanostructures.^33,40^ These techniques make use of a vector network analyzer (VNA) or a combination of MW signal generator, microwave diode and lock-in amplifier. The sample to be investigated is placed on the signal line of CPW or MS and the microwave magnetic field arising from the flow of MW current in the signal line induces spin precession in the sample. On the contrary, RF/WW current is directly injected into the sample in our method and our technique requires only an impedance analyzer to source MW current and record the impedance of the sample. Unlike a network analyzer, the rf impedance analyzer does not need a 50 ohm impedance matching. Even though the maximum attainable frequency is limited to 3 GHz, there are ample opportunities to exploit this technique to other ferromagnetic systems.

## 5. Summary

In summary, we have studied the magnetic field dependence of electrical impedance in the room temperature ferromagnet Pr_0.6_Sr_0.4_MnO_3_ using an impedance analyzer by passing alternating current in the frequency range 1 MHz to 3 GHz directly through the sample. Our magnetoimpedance studies show signature of ferromagnetic resonance which was also confirmed by microwave absorption measurements for multiple frequencies (*f* = 2 to 10 GHz) of microwave electromagnetic field using a commercial broadband ferromagnetic resonance spectrometer. Our line shape analysis indicates a large Gilbert's damping in this sample (*α* = 13.6 × 10^−3^). Our results show that high-frequency magnetoimpedance can be used as a materials characterization tool. Ferromagnetic resonance do find applications in catalysis.^41^ Application of this magnetoimpedance technique to other conducting magnetic oxides may open up a new area of research direction in physical chemistry.

## Conflicts of interest

There are no conflicts to declare.

## Supplementary Material
